# Plasma MicroRNAs in Established Rheumatoid Arthritis Relate to Adiposity and Altered Plasma and Skeletal Muscle Cytokine and Metabolic Profiles

**DOI:** 10.3389/fimmu.2019.01475

**Published:** 2019-06-27

**Authors:** Brian J. Andonian, Ching-Heng Chou, Olga R. Ilkayeva, Timothy R. Koves, Margery A. Connelly, William E. Kraus, Virginia B. Kraus, Kim M. Huffman

**Affiliations:** ^1^Duke Molecular Physiology Institute, Duke University School of Medicine, Durham, NC, United States; ^2^Division of Rheumatology, Department of Medicine, Duke University School of Medicine, Durham, NC, United States; ^3^Laboratory Corporation of America Holdings (LabCorp), Morrisville, NC, United States

**Keywords:** rheumatoid arthritis, microRNA, metabolomics, skeletal muscle, obesity, lipoproteins, disease activity

## Abstract

**Background:** MicroRNAs have been implicated in the pathogenesis of rheumatoid arthritis (RA), obesity, and altered metabolism. Although RA is associated with both obesity and altered metabolism, expression of RA-related microRNA in the setting of these cardiometabolic comorbidities is unclear. Our objective was to determine relationships between six RA-related microRNAs and RA disease activity, inflammation, body composition, and metabolic function.

**Methods:** Expression of plasma miR-21, miR-23b, miR-27a, miR-143, miR-146a, and miR-223 was measured in 48 persons with seropositive and/or erosive RA (mean DAS-28-ESR 3.0, SD 1.4) and 23 age-, sex-, and BMI-matched healthy controls. Disease activity in RA was assessed by DAS-28-ESR. Plasma cytokine concentrations were determined by ELISA. Body composition was assessed using CT scan to determine central and muscle adipose and thigh muscle tissue size and tissue density. Plasma and skeletal muscle acylcarnitine, amino acid, and organic acid metabolites were measured via mass-spectroscopy. Plasma lipoproteins were measured via nuclear magnetic resonance (NMR) spectroscopy. Spearman correlations were used to assess relationships for microRNA with inflammation and cardiometabolic measures. RA and control associations were compared using Fisher transformations.

**Results:** Among RA subjects, plasma miR-143 was associated with plasma IL-6 and IL-8. No other RA microRNA was positively associated with disease activity or inflammatory markers. In RA, microRNA expression was associated with adiposity, both visceral adiposity (miR-146a, miR-21, miR-23b, and miR-27a) and thigh intra-muscular adiposity (miR-146a and miR-223). RA miR-146a was associated with greater concentrations of cardiometabolic risk markers (plasma short-chain dicarboxyl/hydroxyl acylcarnitines, triglycerides, large VLDL particles, and small HDL particles) and lower concentrations of muscle energy substrates (long-chain acylcarnitines and pyruvate). Despite RA and controls having similar microRNA levels, RA, and controls differed in magnitude and direction for several associations with cytokines and plasma and skeletal muscle metabolic intermediates.

**Conclusion:** Most microRNAs thought to be associated with RA disease activity and inflammation were more reflective of RA adiposity and impaired metabolism. These associations show that microRNAs in RA may serve as an epigenetic link between RA inflammation and cardiometabolic comorbidities.

## Introduction

MicroRNAs (miRNAs) are small, non-coding RNAs, ~22 nucleotides long, that regulate post-transcriptional gene expression (1). miRNAs are synthesized by multiple cells and tissues. While miRNA can be passively released upon injury, active release of miRNA in vesicles or exosomes allows miRNA to communicate in autocrine and paracrine fashions. Upon cellular uptake, miRNAs repress protein synthesis by cleaving or blocking translation of target mRNA (2). Individual miRNAs can have one hundred or more mRNA targets across multiple cells and organs systems, while individual mRNA can be bound and repressed by many miRNAs (3). Altered miRNA expression is associated with many disease states (4) and has been implicated in the pathogenesis of autoimmune disease, including rheumatoid arthritis (RA) (5, 6). Thus, miRNAs have been proposed as both RA biomarkers and therapeutic targets (7, 8).

In addition to autoimmune disease, miRNA contribute to the pathogenesis of sarcopenia (9) and obesity (10). However, evaluation of miRNAs in co-morbid disease states, including RA and its associated comorbidities, has received less attention. Despite revolutionary progress in the management of RA inflammation over the past few decades, patients with RA are still at high risk for sarcopenic obesity—decreased skeletal muscle mass with increased fat mass—which contributes to increased risks of disability, cardiovascular disease (CVD), and mortality (11, 12). RA development, severity, and poor treatment responses are tied to obesity (13). Also, RA is associated with sarcopenia, altered skeletal muscle remodeling, and impaired oxidative metabolism (14, 15). While these metabolic impairments in RA are likely driven in part by epigenetic dysregulation (16), it is unclear whether RA-related miRNAs contribute to the RA comorbidities of obesity and altered metabolism. Additionally, it is unclear how complex associations between miRNAs, RA inflammation, obesity, and metabolism impact the potential for miRNAs to be used as biomarkers and therapeutic targets in RA.

In the present study, we measured plasma expression of six miRNAs proposed to be biomarkers of RA inflammation: miR-21 (17), miR-23b (18), miR-27a (19), miR-143 (20), miR-146a (21), and miR-223 (22). Here, we evaluated relationships between miRNA expression and measures of inflammation, adiposity, and altered metabolism. To better understand RA miRNA specific effects, we then compared RA plasma expression of each miRNA to age-, gender-, and race-matched healthy controls. We hypothesized that some miRNAs would reflect RA disease activity, while others would better reflect obesity and metabolic alterations.

## Patients and Methods

### Design and Participants

In a cross-sectional design, patients with RA and matched controls were recruited to participate as previously reported (15). RA subjects (*n* = 48) met American College of Rheumatology 1987 criteria (23); were seropositive (positive rheumatoid factor and/or anti-cyclic citrullinated peptide antibody) or had evidence of erosions on hand or foot imaging; had no medication changes within 3 months of enrollment; and were using ≤ 5 mg prednisone daily. Healthy control subjects (*n* = 23) without a previous diagnosis of inflammatory arthritis or current joint pain were matched to RA subjects by gender, race, age within 3 years, and body mass index (BMI) within 3 kg/m^2^. Subjects were excluded with pregnancy, type 2 diabetes mellitus, or known coronary artery disease. This study complied with the Helsinki Declaration and was approved by the Duke University Institutional Review Board.

### Outcome Measures

All subjects underwent assessments as previously reported and described (15, 24), which included questionnaires, rheumatologic physical exam, fasting phlebotomy, computed tomography (CT) imaging of abdomen and thigh, and *vastus lateralis* muscle biopsies. RA disease activity was measured by the Disease Activity Score in 28 joints (DAS28) with erythrocyte sedimentation rate (ESR) (25). Plasma inflammatory marker and cytokine concentrations were determined by immunoassay (24). CT scan analyses were performed to determine central and muscle adipose and thigh muscle tissue size and tissue density (greater tissue density is indicative of less inter-muscular adipose tissue) (24). Standard Bergstrom needle muscle biopsies were performed on the *vastus lateralis* (26). All plasma and muscle tissue samples were stored at −80°C until analyses.

Analyses of plasma inflammatory markers, plasma, and skeletal muscle metabolic intermediates (acylcarnitines, amino acids, organic acids), and plasma lipoproteins were previously described in detail as follows. Plasma concentrations of inflammatory cytokines interleukin (IL)-1β, IL-6, IL-8, tumor necrosis factor (TNF)-α were measured via enzyme-linked immunosorbent assay (ELISA) (15). Plasma and skeletal muscle acylcarnitines were measured via targeted mass-spectroscopy (15, 27, 28). Skeletal muscle organic acids were measured via gas chromatography–mass spectrometry (GC/MS) (27, 29). Plasma lipoproteins were measured via nuclear magnetic resonance (NMR) spectroscopy (30, 31), while total cholesterol, triglycerides, LDL cholesterol, and HDL cholesterol were measured using standard automated methods.

### Plasma miRNA Expression

miRNA was extracted from plasma samples using the miRCURY RNA Isolation kit—Biofluids from Exiqon (Denmark). Using reverse transcription, cDNAs were generated from miRNAs using the Universal cDNA Synthesis kit from Exiqon (No.203301). The miRNA PCR primer set, based on the SYBR Green miRCURY locked nucleic acids (LNA) detection system from Exiqon, was used according to the manufacturer's instructions to profile plasma expression of hsa-miR-21-5p (YP00204230), hsa-miR-23b-3p (YP00204790), hsa-miR-27a-3p (YP00206038), hsa-miR-143-3p (YP00205992), hsa-miR-146a-5p (YP00204688), and hsa-miR-223-3p (YP00205986). Levels of miRNAs were normalized to a reference hsa-miR-191-5p (YP00204306) and reported as delta cycle threshold (ΔCt), where higher values were equal to more abundant expression.

### Statistical Analysis

**Figure 1:**
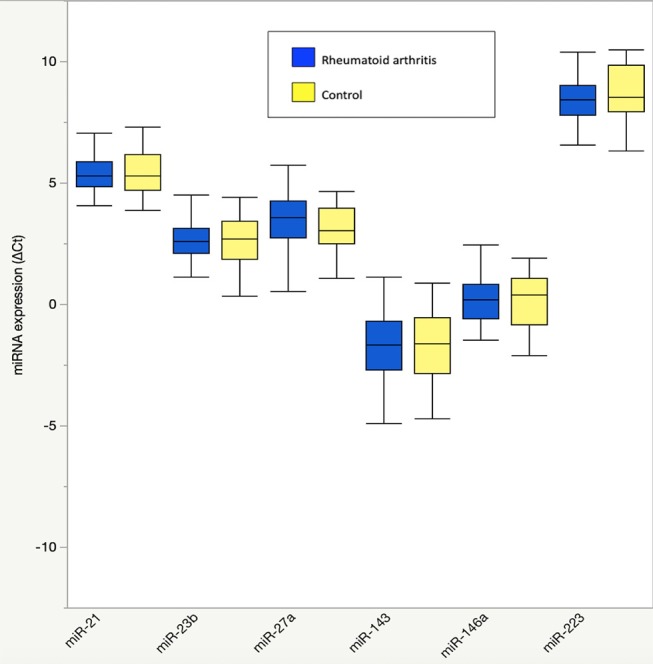
Plasma microRNA expression in rheumatoid arthritis compared to controls. Results are graphically displayed in box plots as medians, inter-quartile ranges (box), and upper and lower 25% of data values, excluding outliers (whiskers) for plasma expression of miR-21, miR-23b, miR-27a, miR-143, miR-146a, and miR-223 in rheumatoid arthritis (*n* = 48) and age-, gender-, and BMI-matched control subjects (*n* = 23). Plasma microRNA expression is reported as delta cycle threshold (ΔCt), where higher values are equal to higher expression. No significant group differences were seen in plasma microRNA expression (*P* > 0.05 for all).

Participant characteristics (Table 1) and plasma miRNAs (Figure 1) were compared in RA vs. control subjects using two sample *t*-tests or Wilcoxon rank-sum tests dependent on whether data conformed to a normal distribution. Analyses of the metabolomic data of the combined RA and control subjects were performed separately for plasma (Table 2) and skeletal muscle (Table 3). Briefly, metabolic intermediates were standardized and reduced using principal components analysis (PCA) with varimax rotation to five factors, each with an eigenvalue >1.0. For each factor, individual metabolites with a factor load >0.4 were reported as factor components. Factor scores were computed for each individual, and correlations between factor scores and clinical assessments were evaluated using Spearman's rho. Strengths of associations for the two groups were compared with Fisher r-to-z transformations (32). All statistical analyses, besides Fisher transformations, were performed using SAS 9.4 (SAS, Cary, NC). Statistical significance was set at *P*-value <0.05. Data are available from the corresponding author upon a reasonable request.

**Table 1:** Participant characteristics.

**Variable**	**Rheumatoid Arthritis (*n* = 48)**	**Controls (*n* = 23)**
Age (years)	55.1 (13.1)	50.7 (13.4)
**Gender**		
Female	33 (68.8%)	17 (73.9%)
**Race**		
Caucasian	34 (70.8%)	17 (73.9%)
African American	13 (27.0%)	6 (26.1%)
Asian/Pacific Islander	1 (0.2%)	0
Disease duration (months)	144.3 (130.8)	N/A
Rheumatoid factor positive	42/47 (89.4%)	N/A
Anti-cyclic citrullinated antibody positive	21/22 (95.6%)	N/A
Erosions present on radiographs	21/38 (55.2%)	N/A
DAS-28 mean	3.0 (1.4)	N/A
BMI (kg/m^2^)	30.3 (7.3)	27.8 (6.0)
Waist circumference (cm)	94.7 (16.5)	89.6 (15.0)

*Data are presented as means (SD) for continuous variables and number (percentages) of participants for dichotomous variables. No significant differences by statistical analyses appeared for all Rheumatoid Arthritis vs. Control comparisons*.

*DAS-28, disease activity score in 28 joints; BMI, body mass index; N/A, not applicable*.

**Table 2:** Plasma metabolite principal component analysis.

**Plasma Factor**	**Name**	**Individual components**
1	Medium-chain acylcarnitines	C10, C8, C12, C12:1, C14:1, C16:1, C10:1, C6, C14, C5-DC, C16:2, C14:2, Pro
2	Long-chain hydroxyl/dicarboxyl acylcarnitines	C18:1, C16, C20-OH/C18-DC, C18:1-OH/C16:1-DC, C16-OH/C14-DC, C20, C18:1-DC, C18:2, C18, C16:1-OH/C14:1-DC, C4-OH, C14-OH/C12-DC, C14:1-OH, C18:2-OH
3	Branched chain amino acids	Met, Leu/Ile, Val, C3, Tyr, C5, Ser, Orn, Phe, His, C5-OH/C3-DC
4	Short-chain dicarboxyl/hydroxyl acylcarnitines	C6-DC/C8-OH, C4-DC/Ci4-DC, C8:1, C10:3, C2, C4/Ci4, C0, C10-OH/C8-DC, C8:1-OH/C6:1-DC, C8:1-DC, C10:2
5	Non-branched chain amino acids	Asx, C5:1, Ala, Arg, Gly, Cit

*Results of principal component analysis from plasma metabolites in combined RA and control groups are presented*.

**Table 3:** Skeletal muscle metabolite principal component analysis.

**Muscle Factor**	**Name**	**Individual components**
1	Skeletal muscle long-chain acylcarinitines, pyruvate	mC20:3, mC18:1, mC22:5, mC18:2, mC22:4, mC20:4, mC20:2, mC18:3, mC20-OH/C18-DC/C22:6, mC20:1, mC16:1, mC16:2-OH/C14:2-DC, mC16, mC22:3, mC18, mPyruvate, mC20, mC22:1
2	Skeletal muscle medium-chain hydroxyl/dicarboxyl acylcarnitines	mC8, mC10, mC12:1, mC14:2, mC14:1, mC10:1, mC6, mC14, mC16:2, mC12, mC14:3, mC14:2-OH/C12:2-DC, mC16:3, mC12:1-OH/C10:1-DC, mC14:1-OH/C12:1-DC, mC4/Ci4, C6-DC/C8-OH:1, mC12:2-OH/C10:2-DC, mC5
3	Skeletal muscle long-chain hydroxyl/dicarboxyl acylcarnitines, lactate, malate, fumarate	mC16-OH/C14-DC, mC18:1-OH/C16:1-DC, mC18:2-OH/C16:2-DC, mC18-OH/C16-DC, mC18:3-OH/C16:3-DC, mC12-OH/C10-DC, mC14-OH/C12-DC, mC16:1-OH/C14:1-DC, mC10-OH/C8-DC, mC20:3-OH/C18:3-DC, mC16:3-OH/C14:3-DC, mC22, mMalate, mC20:1-OH/C18:1-DC, mLactate, mC12:2, mFumarate
4	Skeletal muscle amino acids	mMet, mVal, mPro, mPhe, mGly, mLeu/Ile, mTyr, mHis, mAla, mSer, mOrn, mC0, mArg, mCit, mC5:1, mC3
5	Skeletal muscle short-chain acylcarinitines, citrate, succinate	mC4-DC/Ci4-DC, mC8:1, mC7-DC, mGlx, mC8:1-OH/C6:1-DC, mC2, mC8:1-DC, mC10:2, mCitrate, mC10:3, mC3-DC, mSuccinate

*Results of principal component analysis from skeletal muscle metabolites in combined RA and control groups are presented*.

*m (skeletal muscle)*.

## Results

### Associations Between MicroRNAs and Markers of RA Disease Activity, Inflammation, Adiposity, and Altered Metabolism in RA

In RA, plasma miR-143 was positively related to RA-related systemic inflammation (plasma IL-6 and IL-8) (Table 4), however no miRNA was significantly associated with disease activity (DAS-28; range 0.6–6.4), ESR, or plasma high sensitivity c-reactive protein. miR-21 was associated with less systemic inflammation, greater adiposity, and an altered, pro-atherogenic plasma lipoprotein profile. miR-146a was associated with greater adiposity, pro-atherogenic lipoproteins, and altered plasma (Table 2) and skeletal muscle metabolic intermediates (Table 3). miR-23b and miR-27a were predominantly associated with adiposity while miR-223 was predominantly associated with increased thigh muscle fat, and altered plasma metabolic and lipoproteins profiles, namely small HDL particles, plasma short, and medium chain acylcarnitines and non-branched chain amino acids.

**Table 4:** Plasma microRNAs relationships in rheumatoid arthritis (*n* = 48).

**Variable**	**Plasma microRNAs (ΔCt)**					
	**miR-21**	**miR-23b**	**miR-27a**	**miR-143**	**miR-146a**	**miR-223**
Age (years)	0.39^*^	0.39^*^	0.11	0.03	0.34^*^	0.37^*^
Gender (female)	−0.16	0.01**^†^**	−0.02	0.05	−0.03	0.16**^†^**
Disease activity (DAS28)	−0.04	0.01	−0.01	0.25	0.03	0.09
ESR (mm/hr)	−0.12	0.01	0.08	0.24	0.06	0.07
Plasma hsCRP (mg/L)	−0.19	0.00	0.08	0.08	0.10	0.05
Plasma IL-1β (pg/ml)	–0.31^*^	0.04	−0.12	−0.19	−0.14	0.16
Plasma IL-6 (pg/ml)	−0.07	−0.06	0.11	0.29^*^	0.10	0.14
Plasma IL-8 (pg/ml)	0.09	0.09	0.08	0.33^*^^†^	−0.04	−0.20
Plasma TNF-α (pg/ml)	−0.08	0.03	0.00	0.08	−0.08	−0.02
BMI (kg/m^2^)	0.19	0.23	0.12	0.14	0.41^*^	0.11
Waist circumference (cm)	0.29^*^	0.23	0.28	0.22	0.42^*^	0.07
Visceral adiposity (cm^2^)	0.31^*^	0.42^*^	0.40^*^	0.09	0.49^*^	0.22
Abdominal SQ adiposity (cm^2^)	0.05	0.07	0.03	0.08	0.26	0.00
Thigh IM adiposity (cm^2^)	0.16	0.07	0.00	0.12	0.30^*^	0.02
Thigh SQ adiposity (cm^2^)	0.02	0.03	0.07	0.13	0.28	−0.03
Thigh muscle area (cm^2^)	0.00	−0.07	−0.01	0.03	0.06	−0.09
Thigh muscle density (Hu)	−0.26	−0.25	−0.12	−0.15	–0.35^*^	–0.33^*^
Abdominal liver density (Hu)	0.00	−0.21	−0.23	−0.14	−0.17	−0.24
Plasma metabolite factor 1– medium chain ACs	0.12	0.22	−0.13	0.09	0.22	0.31^*^
Plasma metabolite factor 2– long chain OH/DC ACs	0.14	0.09	0.08	0.05	0.18	0.02
Plasma metabolite factor 3– branched chain AAs	−0.01	−0.05	−0.04	0.04	−0.09**^†^**	−0.19
Plasma metabolite factor 4– short chain DC/OH ACs	0.11	0.23	−0.16	−0.09	0.32^*^	0.37^*^
Plasma metabolite factor 5– non-branched chain AAs	0.12	0.34^*^	0.13	0.04	0.18	0.30^*^
Muscle metabolite factor 1– long chain ACs, pyruvate	−0.28	−0.23	−0.27	−0.17	–0.42^*^^†^	−0.18
Muscle metabolite factor 2– medium chain OH/DC ACs	−0.10	−0.03	0.04	0.02	−0.01	−0.02
Muscle metabolite factor 3– long chain OH/DC ACs, malate, lactate, fumarate	−0.25	−0.18	−0.06	0.13	–0.33^*^	−0.28
Muscle metabolite factor 4– AAs	0.13	−0.03	0.08	−0.05	0.08	0.05
Muscle metabolite factor 5– short chain ACs, citrate, succinate	0.08	−0.08	−0.11	−0.02	−0.01	−0.16
Total cholesterol (mg/dl)	0.13	0.19	0.13	−0.06	0.26	0.12
LDL-cholesterol (mg/dl)	−0.04	0.05	0.07	−0.03	0.14	−0.01
HDL-cholesterol (mg/dl)	0.05	0.06	0.16	−0.07	0.06	0.08
Triglycerides (mg/dl)	0.29^*^	0.28	0.18	0.11**^†^**	0.34^*^	0.20
Plasma Large VLDL-P (nmol/L)	0.29^*^	0.24	0.22	0.06	0.39^*^	0.15
Plasma Small VLDL-P (nmol/L)	−0.03	−0.03	−0.14	−0.05	−0.18	−0.07
Plasma Large LDL-P (nmol/L)	0.09	0.15	0.16	0.08	0.08	0.13
Plasma Small LDL-P (nmol/L)	0.12	0.06	−0.06	0.03	0.22	0.15
Plasma Large HDL-P (μmol/L)	0.06	−0.02	0.02	0.02	−0.05	0.03
Plasma Small HDL-P (μmol/L)	0.20	0.18	0.11	0.01	0.31^*^	0.36^*^

*Data are shown as Spearman correlation coefficients. miR, microRNA; ΔCt, delta cycle threshold; DAS28, Disease Activity Score-28; ESR, erythrocyte sedimentation rate; hsCRP, high sensitivity c-reactive protein; IL, interleukin; BMI, body mass index; SQ, subcutaneous; IM, intramuscular; Hu, Hounsfield units; ACs, acylcarnitines; OH, hydroxyl; DC, dicarboxyl; AAs, amino acids; VLDL, very low density lipoprotein; LDL, low density lipoprotein; HDL, high density lipoprotein; P, particle*.

* ***p<0.05** for Spearman correlation*.

† *p < 0.05 for Fisher r-to-z transformation two-tailed comparisons of RA vs. control (i.e., RA correlation coefficients with opposite directions of magnitude, positive vs. negative, compared to controls)*.

### MicroRNA Associations With Markers of Inflammation and Altered Metabolism Are Different in RA Compared to Controls

There were no differences in RA and control plasma miRNA expression levels (Figure 1), but RA and controls differed in miRNA associations. In controls, miR-143 was negatively correlated with plasma IL-8 (Supplemental Table 1; *r* = −0.34), which significantly differed from the positive association in RA (*r* = 0.33; Fisher r-to-z *P* < 0.05). In controls, miR-146a was positively correlated with plasma branched chain amino acids (Table 2) (Supplemental Table 1; *r* = 0.45) and skeletal muscle long-chain acylcarnitines and pyruvate (Table 3) (Supplemental Table 1; *r* = 0.08), differing significantly from the negative associations in RA (*r* = −0.09 and −0.42, respectively; Fisher r-to-z *P* < 0.05 for both).

## Discussion

In this cohort of established RA, several plasma miRNAs showed unique patterns of association with systemic pro-inflammatory cytokines, adiposity, and impaired metabolism. Among six miRNA selected based on prior associations with RA disease activity and/or inflammation, only miR-143 was reflective of RA systemic inflammation. Rather, plasma miRNAs in our RA cohort associated with measures of adiposity and metabolic alteration. These unique and unexpected associations, along with multiple associations that significantly differed from those of matched controls, highlight the complexity of miRNA functions, especially as they contribute to RA and associated comorbidities.

In contrast to previous reports in the literature (7), we did not find significant associations between inflammation and miR-146a. Inflammation is expected to induce miR-146a expression as part of a feedback mechanism to down-regulate the inflammatory response, including acute inflammation as well as Th1-mediated interferon responses (33–35). We hypothesize that our findings reflect differences in the inflammatory signatures of the RA patients with long-standing disease in our cohort as opposed to those with early, acute inflammatory disease. In our cohort of established RA, miR-146a was instead associated with multiple measures of adiposity as well as plasma short-chain dicarboxyl/hydroxyl acylcarnitines, strong markers of myocardial infarction and coronary artery disease risk (36). Plasma miR-146a was also differentially associated with plasma amino acids, as well as skeletal muscle long-chain acylcarinitines and pyruvate, both key substrates for muscle energy generation, compared to control subjects. These findings are supportive of miR-146a's role in modulating systemic metabolic function, which appears to be altered in RA.

miR-146a mechanistically downregulates TNF-α-induced adipogenesis (37, 38) and oxidative metabolism (39). Our findings suggest that miR-146a expression is appropriate (i.e., increased in response to limit adipogenesis), but is unable to adequately regulate specific metabolic processes due to altered interaction with target mRNAs. Consistent with this hypothesis, miR-146a polymorphisms are not more common in RA (40, 41), but rather RA susceptibility is associated with polymorphisms in known target mRNA binding sites (41).

miRNA alterations likely occur in other RA miRNAs, including miR-143 and miR-223. For example, miR-143 functions to induce inflammation through activation of NF-κB, and other studies show cellular expression of miR-143 is increased in RA synovial tissue (20, 42). In our cohort, miR-143 was positively associated with plasma inflammatory cytokines IL-6 and IL-8 in RA but negatively associated with plasma IL-8 in controls, indicating in RA, the miR-143 pro-inflammatory stimulating response is overactive. In contrast, while miR-223 down-regulates inflammation through multiple mechanisms (43, 44), counterintuitively, in RA, miR-223 expression is increased in multiple sites, including PBMCs, synovial tissue, and plasma (7). In our RA cohort, plasma miR-223 expression did not differ from controls or associate with RA disease activity or inflammation.

miR-223 is also associated with obesity (45) and HDL molecules, which transport miR-223 for lipid metabolic regulatory functions (46). We found RA miR-223 associated with thigh intramuscular fat, small HDL particles, plasma short, and medium chain acylcarnitines and non-branched chain amino acids. Thus, miR-223 alterations may contribute to incomplete systemic beta-oxidation and amino acid catabolism reflecting an obesity-related mitochondrial lipid overload state (47). Further study regarding the effects of these miRNAs on metabolic pathways is warranted.

While this study helps to inform how miRNAs likely exert effects on multiple biologic pathways crucial to RA-associated autoimmunity and impaired metabolism, the findings should be viewed in the context of a few key limitations. First, we did not find any significant differences in expression of candidate plasma miRNA between established RA and control subjects; in contrast, in larger cross-sectional cohorts, there was differential plasma expression of miR-146a and miR-233 between cohorts (7, 48). Whether these findings are the result of the heterogeneity of the RA subjects participating at different investigational sites, low overall RA disease activity, small sample size, or a lack of younger, early RA subjects in our cohort is unclear. Interestingly, previous research shows that epigenetic signatures differ in early vs. longstanding RA (49). Second, the focus of this exploratory study was to identify possible associations between plasma miRNAs and clinical, inflammatory, and metabolic factors to guide further in-depth research; thus, no direct causal pathways were evaluated. Third, we did not choose miRNAs based on microarray studies and thus our analyses were limited to individually measured miRNAs in this study. Finally, we measured miRNA expression only in plasma, and not tissue specific sites, such as immune cells, adipose tissue, or skeletal muscle. We note that quantification of circulating miRNAs may not accurately reflect expression in the tissue (i.e., skeletal muscle) (50). We did not measure the mode of miRNA packaging, either into microvesicles or exosomes, or attached to lipoproteins or other circulating proteins. Knowing the site of miRNA expression, mode of packaging, and target tissue or cellular site of mRNA regulatory function may be helpful in order to better understand these complex miRNA effector pathways.

In conclusion, multiple miRNAs in RA were associated with inflammatory and metabolic pathways in an opposite direction of both expected function and comparative findings in controls. In contrast to previous studies, we found that RA and matched controls had similar amounts of plasma miR-21, miR-23b, miR-27a, miR-143, miR-146a, and miR-223; and only miR-143 was positivity associated with inflammation in RA. Conversely, in RA, miR-146a and miR-223 were prominently associated with age, obesity, plasma and muscle metabolic intermediates, and plasma lipoproteins. Taken together, these findings show in the context of RA, miRNAs influence multiple inflammatory and metabolic pathways across a variety of cells and organ systems. These RA miRNA associations with adipose tissue and metabolic alterations may provide insight into epigenetic connections whereby chronic inflammation leads to common RA comorbidities of obesity and altered metabolism. Further research is needed to clarify the multitude of effects that miRNAs influence in order to utilize miRNAs as diagnostic tools and disease modifying therapies in RA.

## Data Availability

The raw data supporting the conclusions of this manuscript will be made available by the authors, without undue reservation, to any qualified researcher.

## Ethics Statement

This study was carried out in accordance with the recommendations of the Duke University Medical Center Institutional Review Board with written informed consent from all subjects. All subjects gave written informed consent in accordance with the Declaration of Helsinki. The protocol was approved by the Duke University Medical Center Institutional Review Board (IRB no. Pro00064057).

## Author Contributions

BA, CC, VK, WK, and KH conceived and designed the study and experimental approach. KH performed the skeletal muscle biopsies. CC completed the miRNA analyses. OI and TK completed the metabolomic analyses. MC completed the lipoprotein analyses. BA and KH performed statistical analyses. BA wrote the manuscript. All authors contributed to the writing and approval of the final manuscript.

### Conflict of Interest Statement

MC was employed by company LabCorp (Morrisville, NC, USA). The remaining authors declare that the research was conducted in the absence of any commercial or financial relationships that could be construed as a potential conflict of interest.
