# Supplementary material for: Plasma MicroRNAs in Established Rheumatoid Arthritis Relate to Adiposity and Altered Plasma and Skeletal Muscle Cytokine and Metabolic Profiles
Source: Front Immunol. 2019 Jun 27;10:1475. doi: 10.3389/fimmu.2019.01475 (PMC6610455; doi:10.3389/fimmu.2019.01475)
Supplement: Supplementary file 1 [file Table_1.DOCX]

Supplementary Table 1: Plasma microRNAs relationships in healthy controls [n=23]

|  | Plasma microRNAs (ΔCt) | | | | | |
| --- | --- | --- | --- | --- | --- | --- |
| Variable | miR-21 | miR-23b | miR-27a | miR-143 | miR-146a | miR-223 |
| Age (years) | -0.05 | 0.07 | 0.36 | -0.17 | 0.27 | 0.01 |
| Gender (female) | -0.09 | **-0.52*†** | -0.07 | 0.10 | -0.30 | **-0.43*†** |
| ESR (mm/hr) | -0.02 | -0.19 | 0.40 | 0.32 | 0.05 | -0.12 |
| Plasma hsCRP (mg/L) | -0.13 | -0.22 | 0.05 | 0.04 | 0.09 | -0.22 |
| Plasma IL-1β (pg/ml) | -0.26 | -0.12 | 0.17 | -0.26 | -0.12 | -0.11 |
| Plasma IL-6 (pg/ml) | 0.18 | -0.02 | 0.05 | 0.20 | -0.25 | -0.04 |
| Plasma IL-8 (pg/ml) | -0.08 | 0.22 | -0.03 | **-0.34†** | -0.07 | 0.27 |
| Plasma TNF-α (pg/ml) | 0.11 | -0.13 | 0.28 | 0.27 | -0.19 | -0.06 |
| BMI (kg/m^2^) | -0.23 | 0.15 | 0.37 | 0.19 | 0.30 | 0.12 |
| Waist circumference (cm) | -0.14 | 0.25 | 0.20 | 0.00 | 0.25 | 0.24 |
| Visceral adiposity (cm^2^) | 0.17 | 0.35 | 0.28 | -0.29 | 0.37 | 0.31 |
| Abdominal SQ adiposity (cm^2^) | -0.26 | -0.21 | 0.05 | 0.09 | -0.09 | -0.14 |
| Thigh IM adiposity (cm^2^) | -0.14 | 0.13 | 0.40 | 0.16 | 0.26 | 0.13 |
| Thigh SQ adiposity (cm^2^) | -0.42 | -0.41 | 0.12 | 0.30 | -0.20 | -0.38 |
| Thigh muscle area (cm^2^) | -0.08 | 0.23 | 0.07 | 0.14 | 0.14 | 0.19 |
| Thigh muscle density (Hu) | -0.33 | -0.19 | -0.34 | -0.10 | **-0.49*** | -0.23 |
| Abdominal liver density (Hu) | 0.19 | -0.26 | -0.11 | 0.26 | 0.03 | -0.27 |
| Plasma metabolite factor 1 –  medium chain ACs | -0.30 | -0.28 | 0.10 | -0.12 | -0.17 | -0.31 |
| Plasma metabolite factor 2 –  long chain OH/DC ACs | 0.02 | 0.25 | 0.28 | -0.13 | 0.25 | 0.31 |
| Plasma metabolite factor 3 –  branched chain AAs | 0.22 | 0.15 | 0.24 | 0.19 | **0.45*†** | 0.13 |
| Plasma metabolite factor 4 –  short chain DC/OH ACs | -0.25 | 0.06 | 0.02 | -0.16 | -0.03 | 0.03 |
| Plasma metabolite factor 5 –  non-branched chain AAs | 0.04 | -0.05 | 0.20 | 0.10 | 0.12 | -0.03 |
| Muscle metabolite factor 1 –  long chain ACs, pyruvate | 0.39 | 0.06 | 0.03 | 0.31 | 0.08**†** | 0.12 |
| Muscle metabolite factor 2 –  medium chain OH/DC ACs | 0.25 | -0.03 | -0.23 | 0.02 | 0.00 | -0.16 |
| Muscle metabolite factor 3 –  long chain OH/DC ACs, malate, lactate, fumarate | -0.02 | -0.23 | -0.21 | -0.22 | 0.03 | -0.21 |
| Muscle metabolite factor 4 –  AAs | 0.05 | 0.22 | 0.07 | 0.11 | -0.01 | 0.23 |
| Muscle metabolite factor 5 –  short chain ACs, citrate, succinate | -0.08 | 0.07 | -0.31 | -0.23 | -0.25 | 0.20 |
| Total cholesterol (mg/dl) | 0.03 | -0.08 | -0.39 | **-0.42*** | 0.02 | -0.05 |
| LDL-cholesterol (mg/dl) | -0.07 | -0.03 | -0.30 | -0.35 | 0.15 | 0.01 |
| HDL-cholesterol (mg/dl) | 0.27 | -0.16 | -0.19 | 0.05 | -0.27 | -0.15 |
| Triglycerides (mg/dl) | -0.13 | -0.03 | -0.07 | -0.41**†** | -0.06 | -0.03 |
| Plasma Large VLDL-P (nmol/L) | -0.05 | 0.06 | 0.05 | -0.33 | -0.09 | 0.00 |
| Plasma Small VLDL-P (nmol/L) | 0.01 | 0.08 | -0.35 | -0.37 | -0.04 | -0.09 |
| Plasma Large LDL-P (nmol/L) | 0.00 | -0.05 | -0.18 | -0.27 | 0.04 | -0.08 |
| Plasma Small LDL-P (nmol/L) | -0.13 | 0.14 | 0.07 | 0.03 | 0.21 | 0.10 |
| Plasma Large HDL-P (µmol/L) | 0.19 | -0.16 | -0.26 | -0.04 | -0.40 | 0.01 |
| Plasma Small HDL-P (µmol/L) | 0.11 | 0.23 | -0.16 | -0.08 | 0.34 | 0.25 |

Data are shown as Spearman correlation coefficients. miR [microRNA], ΔCt [delta cycle threshold], ESR [erythrocyte sedimentation rate], hsCRP [high sensitivity c-reactive protein], IL [interleukin], BMI [body mass index], SQ [subcutaneous], IM [intramuscular], Hu [Hounsfield units], ACs [acylcarnitines], OH [hydroxyl], DC [dicarboxyl], AAs [amino acids], VLDL [very low density lipoprotein], LDL [low density lipoprotein], HDL [high density lipoprotein], P [particle]

*** p < 0.05** for Spearman correlation.

† p < 0.05 for Fisher r-to-z transformation two-tailed comparisons of RA versus control [ie. RA correlation coefficients with opposite directions of magnitude, positive versus negative, compared to controls].
